# The Smoke Nuisance

**Published:** 1900-10

**Authors:** 


### THE SMOKE NUISANCE.

THE doctors of Cleveland, Ohio, are protesting against the smoke nuisance and cooperating to have it suppressed. The cue is probably taken from the London movement, which has been on foot for several years and has been the occasion of unlimited indulgence in the English national vice—writing letters to the newspapers.

In neither Cleveland nor London nor anywhere else, is this nuisance more immediately apparent, conspicuously evident and altogether intolerable than in Atlanta. Atlanta proudly points to its home manufactories, its hotels and its towering buildings, but it overlooks in its glow of conscious pride, the huge solid columns of reeling black smoke that belch their sulphurous volumes from these objects of prideful consideration, showering sooty, greasy flakes upon the admiring upturned faces and entering the gaping mouths and open nostrils to penetrate the air passages as far as the respiratory waves admit. This nuisance not only fastens itself upon the faces and facial cavities of the citizens, but it is keenly felt in its destructive influence on clothing and books, furniture and anything of a nature that renders it liable to injury by black soot. The average life of a pair of clean cuffs is two hours, of a collar somewhat less than half this time, and finger-nails and ears are never clean. This is not a pretty picture, but, to follow the lead of the marginal note-maker on the novels of the circulating library, "O how true!"

The only good that seems to "issue, proceed and flow" from this otherwise abhorrent infliction is its fostering influence upon the laundry trade and the eye, ear, nose and throat specialists. Of the former there is a flourishing representative on nearly every city block, and by the latter a sneeze may be heard and investigated in any point of the compass and in any corner of the city. They are as the sand on the seashore in number and wear an air of sleek fat prosperity. As we cannot all be laundrymen and nose specialists, we find the smoke nuisance more and more irksome, and are beginning to cry out at the oppression and the pusillanimous spirit of those responsible, who push economy to a degree that is detrimental to the community.

